# Gastric MALT Lymphoma in Plummer-Vinson Syndrome: Broadening the Spectrum of Associated Malignancies

**DOI:** 10.14309/crj.0000000000002041

**Published:** 2026-03-25

**Authors:** Rhett Harmon, Nicholas Cheung, Dorian Mendoza, Payman Fathizadeh, Daniel Cole, Shaun Chandna

**Affiliations:** 1 Department of Medicine, Olive View-UCLA Medical Center, Sylmar, CA; 2 Vatche & Tamar Manoukian Division of Digestive Diseases, David Geffen School of Medicine at UCLA, Los Angeles, CA; 3 Department of Pathology, Olive View-UCLA Medical Center, Sylmar, CA; 4 Division of Gastroenterology, Department of Medicine, Olive View-UCLA Medical Center, Sylmar, CA

**Keywords:** Plummer-Vinson syndrome, iron deficiency anemia, esophageal web, squamous cell carcinoma, MALT lymphoma

## Abstract

Plummer-Vinson syndrome (PVS) is a constellation of symptoms associated with iron deficiency anemia, esophageal web formation, and squamous cell cancer, typically of the esophagus and pharynx. PVS has rarely been associated with gastric cancer. We report a case of a 59-year-old man found to have PVS with gastric mucosa-associated lymphoid tissue lymphoma, an association to our knowledge not previously described in the literature. This case expands the spectrum of malignancies that may be encountered in patients with PVS and highlights the importance of thorough evaluation for malignancy in patients presenting with iron deficiency anemia.

## INTRODUCTION

Plummer-Vinson syndrome (PVS) is a rare disorder characterized by the classic triad of dysphagia, iron deficiency anemia (IDA), and esophageal webs. This syndrome has an associated risk of upper gastrointestinal malignancies, including squamous cell carcinoma of the pharynx and esophagus, and oral cancer to a lesser extent. Those typically at risk include middle-aged White women, with the most prevalent risk factor being iron deficiency, although thyroid disease, rheumatoid arthritis, and celiac disease have also been associated.^1^ PVS is such a rarity that prevalence data remain unknown, yet reported incidence has decreased further with the rise of iron-fortified foods, particularly in developed countries.^1^ However, despite high IDA prevalence in parts of Africa, PVS reports are sparse, indicating factors beyond population iron levels such as diagnostic access, reporting practices, genetics, and autoimmune contributors likely influence observed incidence and detection.^1^ The contribution of PVS to cancer development is poorly understood. We report a case of gastric mucosa-associated lymphoid tissue (MALT) lymphoma occurring in the setting of PVS.

## CASE REPORT

A 59-year-old man with cirrhosis presented with progressive abdominal distention for 1 month, decreased oral intake, dysphagia, and recent 2-unit blood transfusions at an outside hospital. He denied lightheadedness, dizziness, confusion, abdominal pain, diarrhea, hematemesis, or hematochezia. He arrived tachycardic with a heart rate of 116 bpm, but otherwise hemodynamically stable vitals. Physical examination revealed scleral icterus and sublingual jaundice. Laboratory values demonstrated hemoglobin 7.8 (reference: 13.5–16.5 g/dL), mean corpuscular volume 66.0 (82.0–97.0 fL), red cell distribution width 20.7% (12.0–15.0%), international normalized ratio 1.51 (0.87–1.14), aspartate aminotransferase 50 (15–41 U/L), alanine aminotransferase 22 (14–54 U/L), total bilirubin 6.7 (0.1–1.2 mg/dL), direct bilirubin 2.4 (0.1–0.4 mg/dL), and alkaline phosphatase 160 (38–126 U/L). Iron studies were confounded by transfusion at outside hospital (pretransfusion studies not available) showing iron 55 (37–164 mcg/dL), iron saturation 23.6%, total iron binding capacity 242 (261–478 mcg/dL), and ferritin 106 (22–275 ng/mL). Hemoglobin A2 of 1.9% (2.0%–3.2%) was seen on electrophoresis.

**Figure 1.**
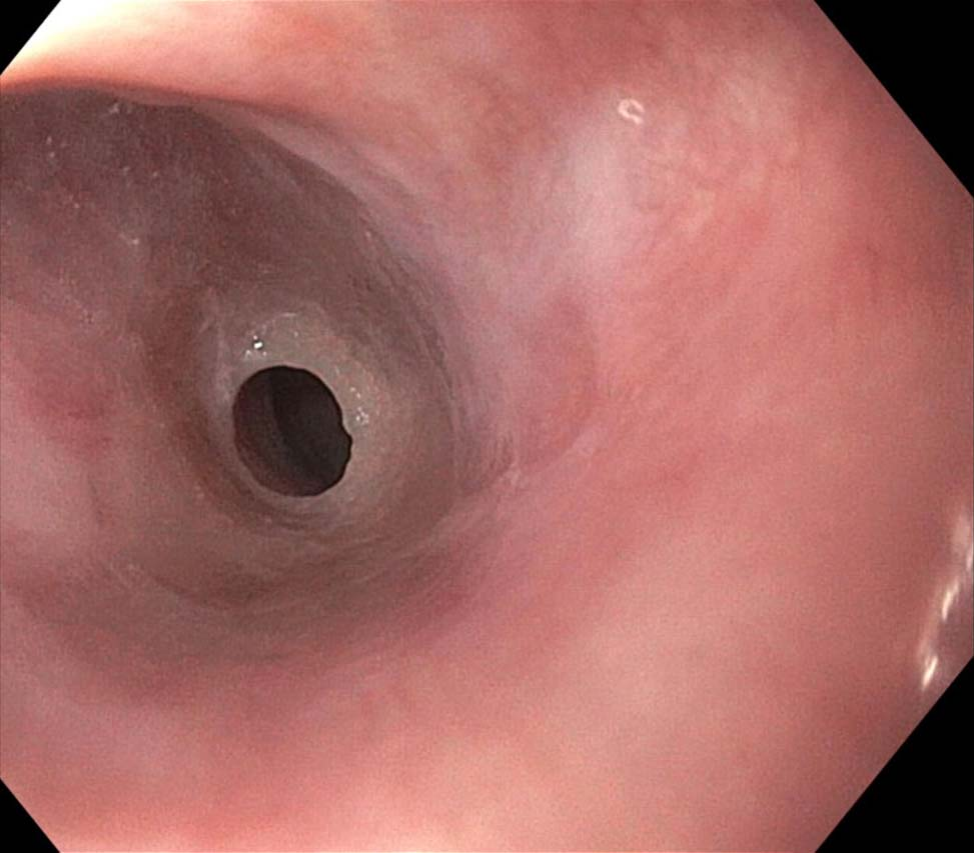
Esophageal web at 15 cm from incisors that was dilated and traversed (image before dilation).

**Figure 2.**
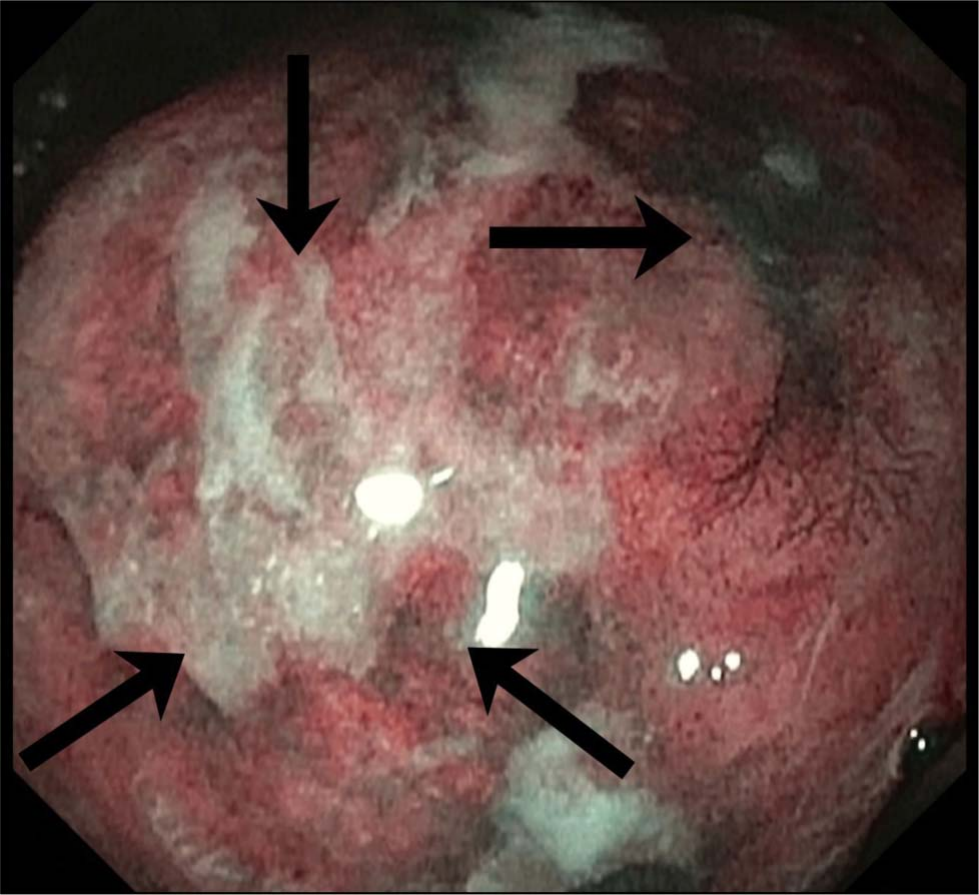
Narrow band imaging of a large 3–4 cm mass on the distal lesser curvature of the gastric body near the angularis, highlighting mucosal vascularity.

**Figure 3.**
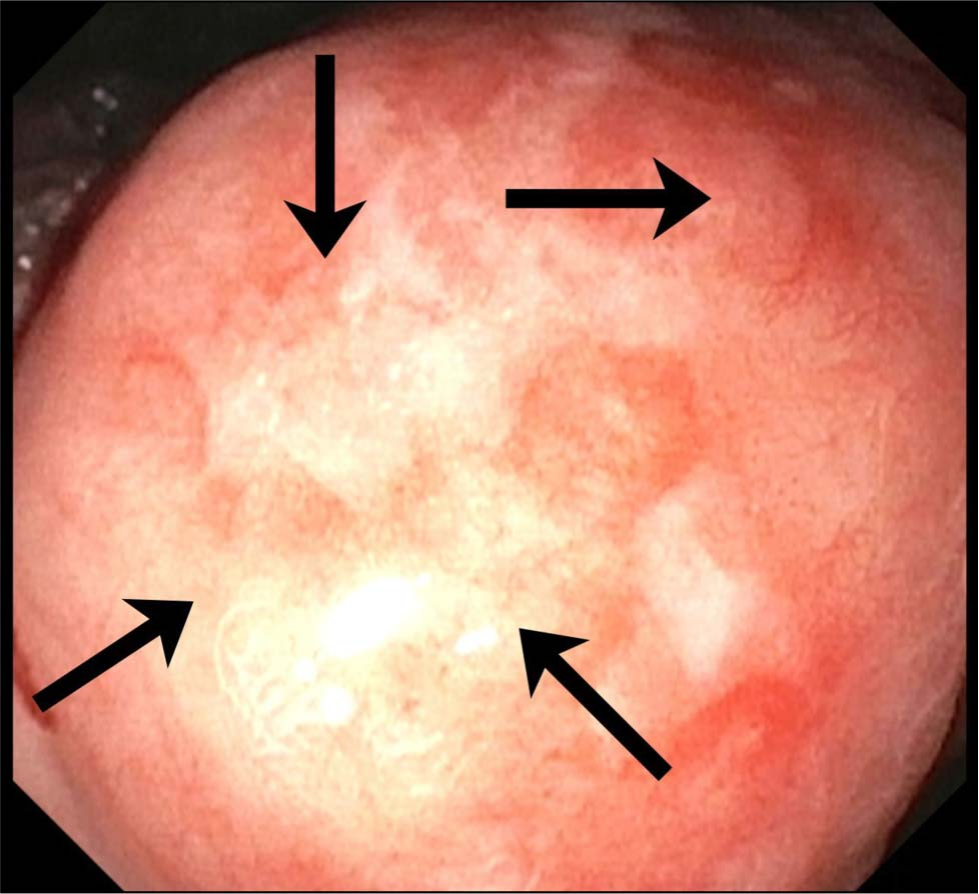
White light imaging of the same lesion, demonstrating friability and spontaneous oozing.

**Figure 4.**
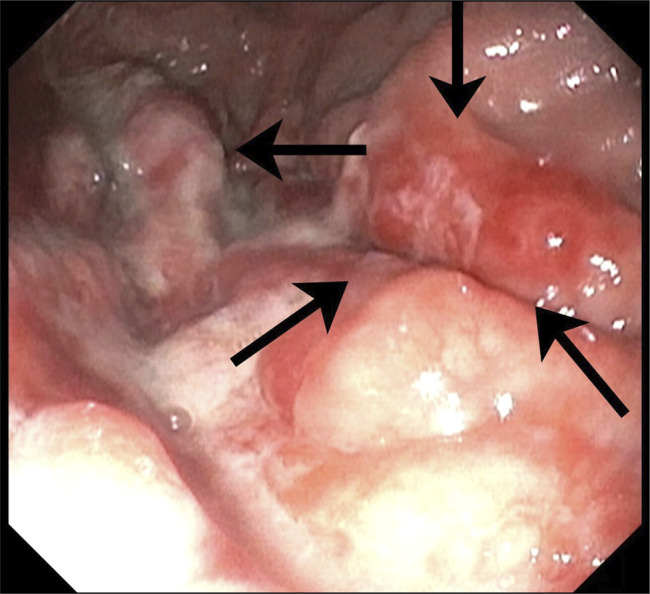
4–5 cm friable mass with vascularity in the fundus.

**Figure 5.**
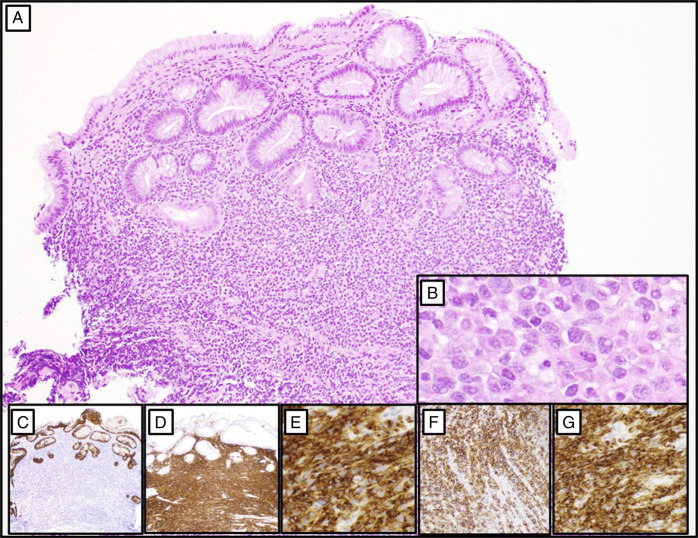
Mass lesion biopsies with immunohistochemical staining (panels A–G). Gastric biopsies demonstrate fragments of mucosa extensively infiltrated by a dense atypical lymphoid population (panel A, 100×). The neoplastic infiltrate consists of small to intermediate-sized lymphocytes characterized by irregular nuclear contours, condensed chromatin, inconspicuous nucleoli, and abundant cytoplasm (panel B, 400×). Immunohistochemical analysis reveals complete loss of native gastric glands, as highlighted by pankeratin staining (panel C, 40×). The infiltrate demonstrates diffuse positivity for B-cell markers including CD20 (panel D, 100×), PAX-5 (panel E, 200×), and immunoreactivity to CD43 (panel F, 200×), and BCL2 (panel G, 200×). The neoplastic cells are negative for CD3, CD5, CD23, BCL1, and BCL6, and exhibit kappa light chain restriction (not shown).

Serologic evaluation for viral hepatitis and autoimmune etiology was negative, other than a nonspecific 1:40 smooth muscle antibody titer and positive hepatitis B core total antibody. Esophagogastroduodenoscopy (EGD) was notable for an esophageal web at 15 cm (Figure 1), a 2 cm pedunculated antral polyp, a 3–4 cm distal lesser curvature mass of the gastric body near the angularis (Figures 2 and 3), and a 4–5 cm friable fundic mass (Figure 4). Random gastric biopsies for *Helicobacter pylori* were not pursued. Biopsies of the antral polyp revealed foveolar hyperplasia. Biopsies of both mass lesions revealed MALT lymphoma with negative immunostaining for *H. pylori* (Figure 5). Fluorescence in situ hybridization analysis demonstrated MALT1 gene rearrangement t(11;18)(q21.2;q21.32). Stool *H. pylori* antigen testing was ordered but not completed. Colonoscopy did not identify lower GI malignancy.

While confounded by the patient's recent transfusion and cirrhosis, the combination of microcytosis and low HbA2 is suggestive of underlying IDA; in conjunction with esophageal web and dysphagia, these findings suggested a diagnosis of PVS.

**Figure 6.**
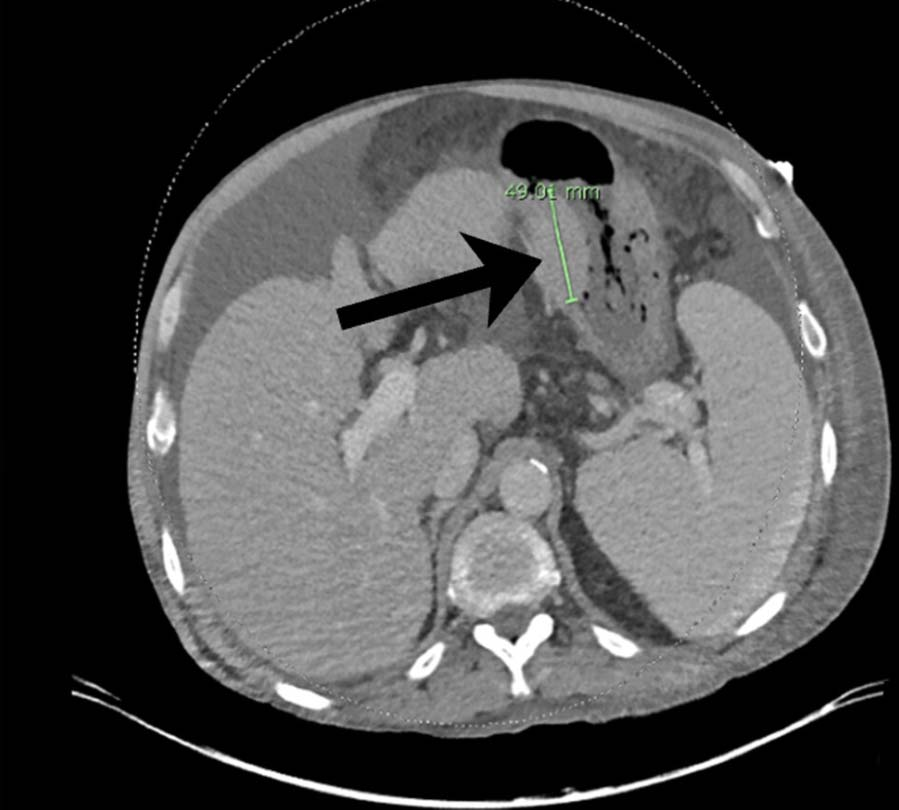
Unilateral thickening of the lesser curvature of the stomach measuring 4.9 cm in length consistent with underlying mass.

Computed tomography of the chest, abdomen, and pelvis revealed a primary gastric lesion (Figure 6). Sequelae of cirrhosis was demonstrated without metastatic disease. Positron emission tomography imaging was ordered but not completed; the patient ultimately underwent radiation therapy for stage 1 (localized) MALT lymphoma with plan for close re-evaluation.

## DISCUSSION

PVS pathophysiology is poorly understood. Chronic iron deficiency may precipitate esophageal muscle atrophy and impaired oxidative repair, resulting in chronic mucosal and epithelial inflammation, predisposing to glossitis and esophagitis with eventual development of esophageal webs.^2–5^ Patients with PVS have an increased risk of malignancy, particularly squamous cell carcinoma, with incidence ranging from 3% to 15%.^1^ Gastric cancer in PVS is rarely reported and is most often gastric adenocarcinoma, with 1 case of synchronous adenocarcinoma and carcinoid (Table 1).

**Table 1.** Gastric cancers associated with PVS in the literature

Citation	Pathology	Treatment	Duration of symptoms	Tumor location
Kitabayashi et al^6^	Gastric adenocarcinoma	Distal partial gastrectomy and endoscopic dilatation	Long-standing PVS (>12 mo)	Antrum (distal stomach)
Kim et al^7^	Gastric adenocarcinoma	Radical total gastrectomy with nodal dissection, splenectomy, and lymphadenectomy	Long-standing PVS (>12 mo)	Cardia and esophagogastric junction
Köklü et al^8^	Undifferentiated gastric adenocarcinoma with metastasis to the liver	Total gastrectomy followed by chemotherapy	Newly symptomatic PVS (<6 mo)	Diffuse involvement of the corpus
Sawalakhe et al^9^	Synchronous adenocarcinoma and carcinoid tumor	Total gastrectomy with local lymphadenectomy	Newly symptomatic PVS (<6 mo)	Distal corpus, greater curvature
Im et al^10^	Poorly differentiated gastric adenocarcinoma	Gastrojejunal bypass and chemotherapy (resection not performed)	Newly symptomatic PVS (<6 mo)	Distal corpus with metastasis to pancreas, cervical spine, lymph nodes, and transverse colon
Jallouli et al^11^	Moderately differentiated gastric adenocarcinoma	Refusal of total gastrectomy and referred to oncology	Long-standing PVS (>12 mo)	Fundus

PVS, Plummer-Vinson syndrome.

By contrast, studies on MALT lymphomas are linked to chronic immune stimulation of infectious or autoimmune origin. Approximately 90% of gastric MALT lymphomas are associated with *H. pylori*, although infections like *Helicobacter heilmannii* have also been implicated.^12^ Site-specific infections such as *Campylobacter jejuni* and other microbes are associated with nongastric MALT lymphomas.^12^ Autoimmune disorders such as Sjögren syndrome and systemic lupus erythematosus are associated with marginal zone and other non-Hodgkin lymphomas.^12^

Evaluation for *H. pylori* is important in MALT lymphoma, as eradication of infection may be curative, though detection can be difficult. In a series of 70 gastric MALT lymphomas, Gu et al^13^ demonstrated 63% histologically *H. pylori*-negative cases, with 89% of these lacking chronic gastritis, suggesting a unique *H. pylori*-negative subgroup or false negative testing. A systematic review including 6,033 patients found *H. pylori*-negative MALT lymphoma in 20.5%, more often in men and associations with proximal lesions, submucosal invasion, and advanced stage.^14^

Fluorescence in situ hybridization is used to detect the t(11;18)(q21.2;q21.32) gene translocation, a hallmark of MALT lymphoma that activates NF-κB signaling and enables antigen-independent B-cell survival.^12^ Multiple studies suggest higher frequency of t(11;18)(q21.2;q21.32) in *H. pylori*-negative disease and predicts resistance to *H. pylori* eradication therapy. Regardless of *H. pylori* status, patients harboring this translocation typically require radiotherapy or systemic chemotherapy.^15,16^ Detection of t(11;18)(q21.2;q21.32) is clinically and prognostically significant as it guides clinical decision-making and therapy.

This case report highlights a rare co-occurrence of PVS and gastric MALT lymphoma. *H. pylori* was not detected on mass lesion immunostaining, though t(11;18)(q21.2;q21.32) was demonstrated which is more often implicated in *H. pylori*-negative disease. Limited assessment for *H. pylori* represents a limitation of this case; evaluation may include stool antigen, breath, and/or biopsy testing (e.g., gastric histology with Sydney protocol and rapid urease testing), with incorporation of a multimodal approach when suspicion remains high.^17^ This patient also had cirrhosis implying a degree of immunocompromise, though cirrhosis itself has not been implicated in MALT lymphoma.^18^ IDA has also been associated with lymphocyte DNA damage and altered immune function; however, evidence linking IDA to malignancy is heterogeneous and not definitive.^19^ Regardless, unexplained IDA can be associated with occult GI bleeding and malignancy, and warrants screening with bidirectional endoscopy in men, postmenopausal women, and asymptomatic premenopausal women.^20^

## DISCLOSURES

Author contributions: Concept and design: R. Harmon, N. Cheung, D. Mendoza, P. Fathizadeh, D. Cole, Shaun Chandna. Drafting of the manuscript: R. Harmon, N. Cheung, S. Chandna. Critical review of the manuscript for important intellectual content: R. Harmon, N. Cheung, D. Mendoza, P. Fathizadeh, D. Cole, S. Chandna. Pathology images: P. Fathizadeh. Supervision: D. Cole, P. Fathizadeh, S. Chandna. R. Harmon is the article guarantor.

Financial support: None to report.

Conflicts of interest: Shaun Chandna has received research funding from Arrowhead Pharmaceuticals, Lipocine, Calliditas Therapeutics (formerly Genkyotex), and Mirum Pharmaceuticals. Shaun Chandna is on the Speaker Bureau and has consulted for Madrigal Pharmaceuticals and has consulted for Mirum Pharmaceuticals and Ipsen. The other authors have no disclosures to report.

Informed consent was obtained for this case report.

Previous presentation: This case was presented as a poster abstract at the American College of Gastroenterology 2025 Annual Scientific Meeting on October 28, 2025 in Phoenix, Arizona.

## ABBREVIATIONS:

EGD: esophagogastroduodenoscopy
GI: gastrointestinal
H. pylori: Helicobacter pylori
IDA: iron deficiency anemia
MALT: mucosa-associated lymphoid tissue
MALT1: mucosa-associated lymphoid tissue lymphoma translocation protein 1
PVS: Plummer-Vinson syndrome
